# A Systematic Review of Worldwide Consumption of Ultra-Processed Foods: Findings and Criticisms

**DOI:** 10.3390/nu13082778

**Published:** 2021-08-13

**Authors:** Mirko Marino, Federica Puppo, Cristian Del Bo’, Valentina Vinelli, Patrizia Riso, Marisa Porrini, Daniela Martini

**Affiliations:** Department of Food, Environmental and Nutritional Sciences (DeFENS), Università degli Studi di Milano, 20123 Milan, Italy; mirko.marino@unimi.it (M.M.); federica.puppo@studenti.unimi.it (F.P.); valentina.vinelli@unimi.it (V.V.); patrizia.riso@unimi.it (P.R.); marisa.porrini@unimi.it (M.P.); daniela.martini@unimi.it (D.M.)

**Keywords:** dietary habits, dietary intake, human health, NOVA group, processing, food technology, food consumption

## Abstract

A debate is ongoing on the significance and appropriateness of the NOVA classification as a tool for categorizing foods based on their degree of processing. As such, the role of ultra-processed food (UPF) on human health is still not completely understood. With this review, we aimed to investigate the actual level of consumption of UPF across countries and target populations to determine the impact in real contexts. Suitable articles published up to March 2021 were sourced through the PubMed and SCOPUS databases. Overall, 99 studies providing data on the level of UPF consumption expressed as the percentage of total energy intake were identified, for a total of 1,378,454 participants. Most of them were published in Brazil (*n* = 38) and the United States (*n* = 15), and the 24 h recall was the most-used tool (*n* = 63). Analysis of the results revealed that the United States and the United Kingdom were the countries with the highest percent energy intake from UPF (generally >50%), whereas Italy had the lowest levels (about 10%); the latter was inversely associated with adherence to the Mediterranean diet. High variability was also observed based on sex, age, and body mass index, with men, young people, and overweight/obese subjects generally having higher levels of consumption compared to older subjects. Overall, our findings underline the large differences in UPF intake. Since most of the observations derived from studies conducted with food questionnaires are not specifically validated for UPF, further efforts are essential to confirm the results previously obtained and to investigate further the association between UPF consumption and health status, also considering the actual contribution within different dietary patterns, which has been less investigated to date.

## 1. Introduction

Food processing includes all the processes needed to transform raw or harvested foodstuffs into new products, ensuring their safety, palatability, and shelf-life [1]. However, in recent decades, food processing has drastically changed to address consumer preferences. The demand for food items with longer shelf-life and improved palatability has led to other natural or artificial ingredients being added to processed foods, which may, to some extent, impact the nutritional quality of these foods, which are often characterized by high sugar, fat, and salt contents [2]. For this reason, interest is growing in elucidating whether the high consumption of these foods negatively impacts diet quality and, in turn, human health.

One of the main limits to the evaluation of the role and impact of food processing on health status is the lack of a proper definition and classification of “processed food”. In 2009, the European Prospective Investigation into Cancer and Nutrition (EPIC) [3] proposed a classification of foods into three main categories: highly processed, moderately processed, and non-processed foods. A Brazilian group of researchers coordinated by Monteiro instead proposed the NOVA classification, which classifies foods into four main categories based on their degree of processing, without providing any indication of their nutritional content: (i) unprocessed and minimally processed foods such as fruit and vegetables, milk, eggs, and meats; (ii) processed culinary ingredients, including oils, butter, lard, sugar, and salt; (iii) processed foods such as canned fish or legumes, typically produced by adding salt, oil, sugar, or other substances from group 2 to group 1 foods, and using preservation methods such as canning and bottling; and (iv) ultra-processed foods (UPF) [4]. The latter comprises formulations of ingredients, mostly for exclusive industrial use, produced through a series of industrial processes that, for instance, include the fractioning of whole foods into substances, the assembly of unmodified and modified food substances, and the frequent use of cosmetic additives, often added to improve the sensory characteristics of the final product [5]. UPF include, amongst others, carbonated soft drinks; sweet, fatty, or salty packaged snacks; candies; ice creams; pastries; margarines; and many others.

The Siga classification further attempts to categorize foods based on processing, which was developed by combining the four holistic NOVA groups with four additional new reductionist subgroups that consider the impact of processing on the food/ingredient matrix; the contents of added salt, sugar, and fat; the nature and number of markers of ultra-processing; and the levels of at-risk additives [6]. 

Despite the different types of food classification systems based on the degree of processing, the NOVA classification is the most widely used worldwide by researchers. Since it was proposed, the NOVA classification has been used in many epidemiological studies to investigate the association between the levels of UPF consumption and diet quality [7,8] and/or the potential effect of UPF consumption on human health. For instance, a narrative review summarized the main findings from observational studies reporting a higher risk of all-cause mortality with high consumption of UPF [9]. In another narrative review, high UPF consumption was associated with an increased risk of all-cause mortality including cardiovascular diseases, coronary heart diseases, cerebrovascular diseases, cancer risk, and numerous other metabolic diseases [10]. More recently, a systematic review and meta-analysis of observational studies highlighted that increased UPF consumption is associated with a worse cardiometabolic risk profile and a higher risk of cardiovascular diseases and all-cause mortality [11]. Furthermore, the results of a randomized controlled trial showed that a two-week intervention with a diet rich in UPF consumed ad libitum resulted in significantly increased weight gain amongst American adults compared to a diet of unprocessed foods [12].

The criteria used in these types of classification are often ambiguous, only based on a chaotic conception of processing that is not only related to technical processes [13]. Notably, the dietary habits of different populations may vary widely based on tradition, culture, and individual characteristics; in turn, the consumption of UPF in terms of type and amount may differ across target populations and countries. 

Based on these premises, understanding the implications of increasing UPF consumption for global human health is of upmost importance, together with the identification of the cause-effect relationship. In order to pursue this important goal, it is first of all crucial to estimate/quantify the amount of UPF consumed adequately. The aim of the present review was to investigate the levels of UPF consumption systematically—as defined by the NOVA system—across countries around the world. The summarized information will be useful to provide a clear overview of the actual consumption of UPF in different countries, of their contribution to total energy intake also in diverse target groups (i.e., children, adolescents, adults), but also to bring out possible criticisms in the estimation of UPF intake and to underline eventual gaps that need to be considered in future studies.

## 2. Materials and Methods

### 2.1. Search Strategy and Study Selection

This study was conducted according to the Preferred Reporting Items for Systematic Reviews Meta-Analysis (PRISMA) guidelines. To identify pertinent articles, we searched the PubMed and SCOPUS databases up to March 2021 using the combination of the following keywords: (“ultraprocessed food*” OR “ultra-processed food*” OR “NOVA system” OR “NOVA classification” OR “minimally processed food”) AND (“intake” OR “consumption”). Reference lists of included manuscripts and relevant reviews were examined for any possible unidentified study. 

Studies were included if they: (i) provided information on UPF consumption expressed as the percentage of energy for each NOVA category with respect to total dietary intake; (ii) provided information for the general population or for at least one target group; (iii) were published in the English language. Conversely, studies were excluded if they: (i) were published before 2009; (ii) did not use the NOVA classification; (iii) were systematic reviews or meta-analyses; (iv) expressed UPF intake as tertiles/quartiles without providing information about the net consumption.

### 2.2. Data Extraction and Presentation

Data from included papers were extracted by two reviewers (M.M. and F.P.). Any discrepancy between the reviewers was resolved through consultation with a third independent author (C.D.B) to achieve consensus. The following information is reported: (i) authors and year of publication; (ii) country; (iii) characteristics of the population under study; (iv) method of dietary assessment; (v) results expressed as a percentage of energy from UPF compared to total energy intake. Studies are summarized in different tables showing the percentage of energy intake (TEI) from UPF compared to total energy intake, considering the main factors such as age, sex, and BMI. Data are reported as the mean or median value followed by the standard deviation (SD), standard error of the mean (SEM), confidence interval (CI), or interquartile range (IQR). When available, the statistical significance is reported.

## 3. Results

### 3.1. Study Selection

**Figure 1:**
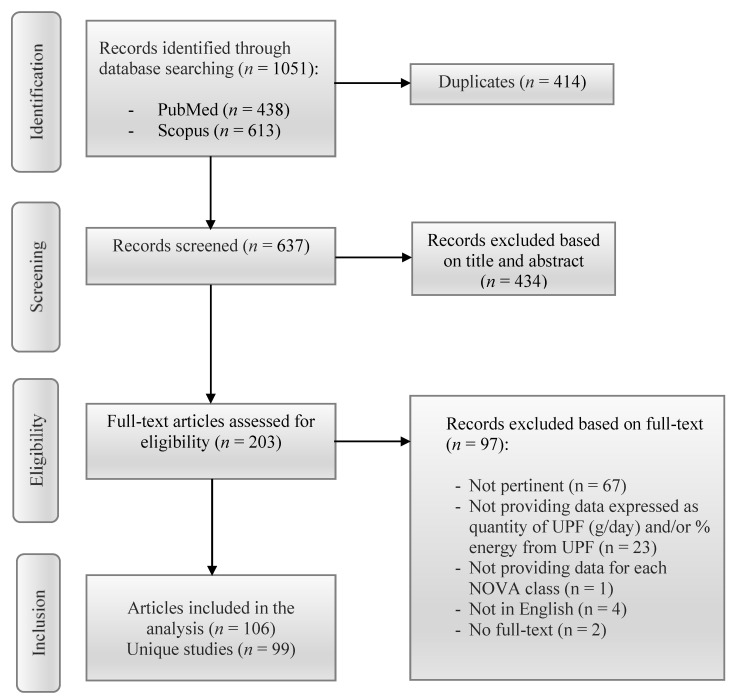
PRISMA flow diagram of the included studies.

A total of 1051 studies, conducted between 2009 and 2021, were identified using the PubMed and SCOPUS databases. After eliminating 414 duplicates, 434 articles were excluded based on the titles and abstracts, and 97 studies were removed based on the full-text assessment. A total of 106 articles met the inclusion criteria so were included in the final analysis. Among these, five studies led to two different articles each, which were therefore considered as unique studies. Thus, at the end of the process, a total of 99 unique studies were included (Figure 1). 

### 3.2. Characteristics of the Studies 

Among the 99 studies, a total of 1,378,454 subjects were considered, with the number of subjects ranging from 40 [14] to 110,260 [15]. On average, the age of participants was 38.9 years, although half of the studies did not report this information. Fifteen studies focused only on children [16,17,18,19,20,21,22,23,24,25,26,27,28,29,30], seven on adolescents [31,32,33,34,35,36,37], five on pregnant women [38,39,40,41,42], and three on older subjects [43,44,45], whereas others focused on an adult population or on ≥2 target groups. Overall, 58.8% of participants were women.

**Figure 2:**
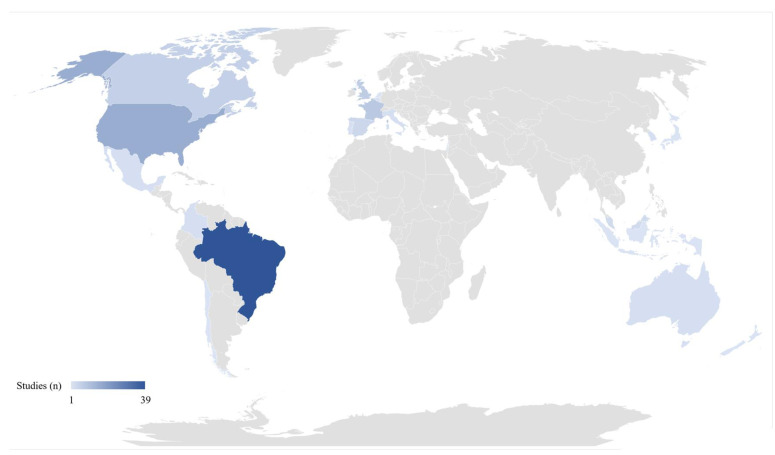
Countries in which studies on consumption levels for UPF were performed; the darker the blue, the higher the number of studies conducted.

The number of studies performed in different countries is reported in Figure 2. As shown, Brazil had the highest number of studies (*n* = 38), followed by the United States (*n* = 15), France (*n* = 8), the United Kingdom (*n* = 6), Canada (*n* = 6), and Spain (*n* = 4). Fewer studies were performed in Malaysia, (*n* = 3), and in other countries such as Mexico, Italy, Australia, Korea, and Portugal (*n* = 2); only one study each was conducted in Chile, Japan, Indonesia, Lebanon, Israel, the Netherlands, Colombia, Belgium, and New Zealand.

Regarding the method of dietary assessment, the 24 h recall was the most-used tool (*n* = 62), followed by food-frequency questionnaires (FFQs) (*n* = 28) and food diaries (*n* = 10).

### 3.3. Levels of UPF Intake

The levels of consumption of UPF shown in the retrieved studies are reported in Table 1, while results stratified for specific target groups are provided in Table 2, Table 3 and Table 4.

The data revealed a high variability in terms of the percentage (%) of energy provided by the consumption of food belonging to group 4 based on the NOVA classification. Similar findings were observed both intra- and inter-country. The highest levels of consumption were observed in the United States and the United Kingdom with the percent of energy intake generally higher than 50% with respect to TEI [18,19,36,38,46,47,48,49,50,51,52,53,54]. Conversely, the lowest levels were observed in Italy, in which the two studies identified reported about 10% energy obtained from UPF [55,56].

Regarding the variability within the same country, Brazil showed levels of UPF consumption ranging from 7.7% in 64 subjects aged 25–57 years [57] to 51.2% of total energy intake in over 4200 subjects [58]. A lower variability was identified in studies performed in the United States, with levels of intake ranging from 50% to 70%, with the only exception being a study observing an energy intake of 35.5% in almost 92,000 subjects aged 55–74 years [59].

**Table 1:** Characteristics of the selected studies (*n* = 106) and the level of consumption of ultra-processed foods (UPF) expressed as the percent of energy provided by UPF intake with respect to total energy intake (TEI).

Author (Year)	Country	Study Population	Assessment of UPF Intake	Results on UPF Consumption
Oliveira et al., (2021) [34]	Brazil	*n* = 462 adolescents (53.5% M) (mean age: 13.1 ± 1.5 years; mean BMI: nd)	Recall 24 h	31.9% of TEI SD or SEM: nd
Graciliano et al., (2021) [40]	Brazil	*n* = 295 pregnant women (100% W) (mean age: 23.7 years; mean BMI: nd)	2 Recall 24 h	22.2% of TEI SD or SEM: nd
Silva et al., (2021) [42]	Brazil	*n* = 42 pregnant women with pregestational diabetes mellitus (100% women) (mean age: 31.5 ± 5.8 years; mean BMI: nd)	FFQ	Data nd (see Supplementary Table S1)
Rocha et al., (2021) [32]	Brazil	*n* = 71,533 adolescents (55.5% W) (mean age: nd, range 12–17 years; mean BMI: nd)	Recall 24 h (*n* = 1626 foods and drinks)	28% (95% CI 27.80–28.15) of TEI
Costa et al., (2021) [60]	Brazil	*n* = 3128 children and 3454 adolescents (51.9% M) (mean age: nd, range 6–11 years; mean BMI: nd)	FFQ	Data nd (see Table 3)
Da Silva et al., (2021) [61]	Brazil	*n* = 670 adults (50.1% M) (mean age: nd, range 20–59 years; mean BMI: nd)	Recall 24 h	24.6 ± 1.32% of TEI
Melo et al., (2021) [31]	Brazil	*n* = 804 adolescents (57.7% W) (mean age: 16.1 ± 1.2 years; mean BMI: nd)	2 Recall 24 h	45.9% (95% CI; 45.1–46.7) of TEI
Scaranni et al., (2021) [62]	Brazil	*n* = 8171 adults (% M/W ND) (mean age: 49 years (35–74 years); mean BMI: nd)	FFQ (116 items)	25.2 ± 9.6% (14.5% for low UPF consumption, up to 35.4% for high consumption) of TEI
Oliveira et al., (2020) [17]	Brazil	*n* = 164 overweight + obese children (59.1% F) (mean age: 8.6 ± 0.8 years; mean BMI: nd)	3 Recall 24 h (3 non-consecutive days, one of them on the weekend)	43.7 ± 13% (95% CI: 41.7–45.7) of TEI
Cattafesta et al., (2020) [63]	Brazil	*n* = 740 farmer adults (51.5% M) (mean age: nd, ≥18 years; mean BMI: nd)	3 Recall 24 h	17.7 ± 10.8% of TEI
Paulino et al., (2020) [41]	Brazil	*n* = 175 high-risk pregnant women (100% W) (mean age: 31.1 ± 6 years; mean BMI: 32.2 ± 7.8 kg/m^2^)	3 Recall 24 h	25.5% of TEI SD or SEM: nd
Viola et al., (2020) [35]	Brazil	*n* = 1525 adolescents (52.9% F) (mean age: nd, range 18–19 years; mean BMI: nd)	FFQ (106 items)	37% of TEI SD or SEM: nd
Lacerda et al., (2020) [23]	Brazil	*n* = 322 children (53.4% F) (mean age: 9.8 ± 0.5 years; mean BMI: nd)	2 Recall 24 h (2 non-consecutive days of the week)	25.2% (95% CI: 23.61–26.83) of TEI
Souza et al., (2020) [64]	Brazil	*n* = 921 adults (55.8% M) (mean age: 38 ± 17.7 years; mean BMI: 27.5 kg/m^2^)	Recall 24 h	20.6% of TEI SD or SEM: nd
Leffa et al., (2020) [24]	Brazil	*n* = 308 children (52% M) (mean age: 3.2 ± 0.1 years and 6.3 ± 0.2 years; mean BMI: nd)	2 Recall 24 h (non-consecutive days)	Data nd (see Table 3)
Smaira et al., (2020) [65]	Brazil	*n* = 56 women with rheumatoid arthritis (100% W) (mean age: 62.5 ± 7.9 years; mean BMI: 28.4 ± 5.1 kg/m^2^)	3 Recall 24 h (3 non-consecutive days)	18.1 ± 11.8% of TEI
Canhada et al., (2020) [66]	Brazil	*n* = 11827 adult + older subjects (55% F) (mean age: 51.3 ± 8.7 years (35–74 years); mean BMI: 26.8 ± 4.6 kg/m^2^)	FFQ (114 items)	24.6 ± 9.6% of TEI
Silverio et al., (2019) [67]	Brazil	*n* = 120 children + adolescents (53.3% W) (mean age: 11.7 ± 2.8 years; mean BMI: nd)	Recall 24 h	24.2 ± 17.9% of TEI
Sousa et al., (2020) [68]	Brazil	*n* = 2499 adolescents (52.3% W) (mean age: 18–19 years; mean BMI: nd)	FFQ (106 items)	35.8 ± 13.1% of TEI
Longo et al., (2020) [69]	Brazil	*n* = 74 subjects with atherosclerosis (66.2% M) (mean age: 60.7 ± 1.1 years; mean BMI: 28.7 ± 0.5 kg/m^2^) *	Recall 24 h	35.1% of TEI SD or SEM: nd
Rezende-Alves et al., (2020) [70]	Brazil	*n* = 1221 adults (76.1% W) (mean age: 35.2 ± 9.1 years; mean BMI: nd)	FFQ (144 items)	25.8 ± 11% of TEI
Fonseca et al., (2019) [25]	Brazil	*n* = 403 children (55.1% M) (mean age: 71.8 ± 12 months; mean BMI: nd)	3-day food diary (1 day of the weekend)	38 ± 1% of TEI
Da Conceição et al., (2019) [57]	Brazil	*n* = 64 adults (64.1% W) (mean age: nd, range 25–57 years; mean BMI: nd)	Recall 24 h	7.7% of TEI SD or SEM: nd
Fortins et al., (2019) [71]	Brazil	*n* = 120 children + adolescents (53.3% W) (mean age: 11.7 ± 2.8 years; mean BMI: nd)	Recall 24 h	24.3 ± 17.9% of TEI
Ferreira et al., (2019) [26]	Brazil	*n* = 206 children + adolescents (53% W) (age > 10 years; mean BMI: nd)	Recall 24 h	31% of TEI SD or SEM: nd
Gomes et al., (2019) [39]	Brazil	*n* = 353 pregnant women (100% W) (mean age: nd; mean BMI: nd)	2 Recall 24 h	24.6% of TEI SD or SEM: nd
Enes et al., (2019) [37]	Brazil	*n* = 200 adolescents (56% F) (mean age: nd, range 10–18 years; mean BMI: nd)	FFQ	50.6 ±1.0% * of TEI
Silva et al., (2018) [72]	Brazil	*n* = 8977 adult + older subjects (51.9% W) (mean age: nd, range 35–64 years; mean BMI: nd)	FFQ (114 items)	22.7% of TEI SD or SEM: nd
Simões et al., (2018) [73]	Brazil	*n* = 14378 adult + older subjects (54.2% W) (mean age: nd, range 35–74 years; mean BMI: nd)	FFQ (114 items)	22.7% of TEI SD or SEM: nd
Louzada et al., (2018) [74]	Brazil	*n* = 32,898 subjects (% M/W: nd) (mean age: nd, ≥10 years; mean BMI: nd)	2 Recall 24 h	20.4% of TEI SD or SEM: nd
Bielemann et al., (2018) [28]	Brazil	*n* = 3427 children (51.9% M) (mean age: 6 years; mean BMI: nd)	FFQ (54 items)	40.3 ± 11.7% of TEI
D’Avila et al., (2017) [33]	Brazil	*n* = 784 adolescents (57.4% W) (mean age: 15.2 ± 1.3 years; mean BMI: nd)	FFQ (90 items)	49.2% of TEI SD or SEM: nd
Batalha et al., (2017) [27]	Brazil	*n* = 1185 children (51.2% M) (mean age: nd, range 15–35 mo.; mean BMI: nd)	Recall 24 h	24.5% of TEI SD or SEM: nd
Karnopp et al., (2017) [29]	Brazil	*n* = 770 children (52% M) (mean age: nd, range 0–72 mo; mean BMI: nd)	Recall 24 h	32% of TEI SD or SEM: nd
Bielemann et al., (2015) [58]	Brazil	*n* = 4202 adults (51.4% M) (mean age: 21.8 years; mean BMI: nd)	FFQ	51.2% (95% CI: 50.8–51.6) of TEI
Sparrenberger et al., (2015) [30]	Brazil	*n* = 204 children (50% girls) (mean age: 5.9 ± 2.5 years; mean BMI: nd)	2 Recall 24 h	47 ± 1.1% * of TEI
Louzada et al., (2015a) [75]	Brazil	*n* = 32,898 subjects (% M/F: nd) (mean age: nd, ≥10 years; mean BMI: nd)	2 Recall 24 h	21.5% of TEI SD or SEM: nd
Louzada et al., (2015b) [76]	Brazil	*n* = 30,243 subjects (50.2% W) (mean age: nd, ≥10 years; mean BMI: nd)	2 Recall 24 h	29.6% of TEI SD or SEM: nd
Baraldi et al., (2021) [77]	USA	*n* = 24,505 subjects (% M/F: nd) (mean age: nd, ≥1 years; mean BMI: nd)	Recall 24 h	57.9% of TEI SD or SEM: nd
Bidinotto et al., (2021) [78]	USA	*n* = 5720 adults (50.8% W) (mean age: 39.6 ± 0.4 years; mean BMI: nd)	2 Recall 24 h	56.9 ± 0.5% of TEI
Zhong et al., (2021) [59]	USA	*n* = 91,891 adults + older subjects (% M/W: nd) (mean age: nd, range 55–74 years; mean BMI: nd)	FFQ (137 items)	35.5 ± 16.6% of TEI
Yang et al., (2020) [79]	USA	*n* = 12,640 adults (50.2% W) (mean age: 49.7 years; mean BMI: 29.4 kg/m^2^)	2 Recall 24 h	54.5% of TEI SD or SEM: nd
Zheng et al., (2020) [80]	USA	*n* = 13,637 adults + older subjects (50.1% W) (mean age: nd, >20 years; mean BMI: nd)	Recall 24 h	55% of TEI SD or SEM: nd
Gupta et al., (2020) [81]	USA	*n* = 755 adults (82.1% W) (mean age: nd, range 21–59 years; mean BMI: nd)	FFQ (126 items)	59.7 ± 10.7% of TEI
Smiljanec et al., (2020) [14]	USA	*n* = 40 adults 62.5% W) (mean age: 27 ± 1 years; mean BMI: 23.6 ± 0.5 kg/m^2^)	3-day food diary	50 ± 2.4% of TEI
Zhang et al., (2021) [82]	USA	*n* = 11,246 adults (51% W) (mean age: 44.6 ± 0.4 years; mean BMI: nd)	2 Recall 24 h	55.4% of TEI SD or SEM: nd
Martinez Steele et al., (2020) [51]	USA	*n* = 9416 subjects (% M/W: nd) (mean age: nd, ≥6 years; mean BMI: nd)	2 Recall 24 h	58% ± 0.5% * of TEI
Neri et al., (2019) [18]	USA	*n* = 9469 children + adolescents (% M/F: nd) (mean age: nd, range 2–19 years; mean BMI: nd)	2 Recall 24 h	64.6% of TEI SD or SEM: nd
Martínez-Steele et al., (2019) [83]	USA	*n* = 6385 adults + older subjects (% M/W: nd) (mean age: nd, ≥20 years; mean BMI: nd)	2 Recall 24 h	55.5 ± 0.5%* of TEI
Baraldi et al., (2018) [49]	USA	*n* = 23,847 subjects (% M/W: nd) (mean age: nd, ≥2 years; mean BMI: nd)	2 Recall 24 h	58.5 ± 0.3% * of TEI UPF consumption for years: 2007–2008 years = 57.6% of TEI 2009–2010 years = 58.9% of TEI 2011–2012 years = 59.7% of TEI
Juul et al., (2018) [52]	USA	*n* = 15,977 adults (50.6% W) (mean age: 41.9 ± 0.2 years; mean BMI: 28.9 ± 0.1 kg/m^2^)	2 Recall 24 h	56.1 ± 25.4% of TEI
Rohatgi et al., (2017) [38]	USA	*n* = 45 pregnant women (100% W) (mean age: nd; mean BMI: nd)	FFQ	54.4 ± 13.2% of TEI
Martínez-Steele et al., (2016) [50]	USA	*n* = 9317 subjects (% M/W: nd) (mean age: nd, ≥1 years; mean BMI: nd)	2 Recall 24 h	57.9% of TEI SD or SEM: nd
Calixto Andrade et al., (2021) [84]	France	*n* = 2642 adults + older subjects (63.3% W) (mean age: nd, ≥18 years; mean BMI: nd)	3 Recall 24 h (week days and weekend days)	31.1% (95% CI: 30.3, 31.9) of TEI
Gehring et al., (2020 and 2021) [85,86]	France	*n* = 21,212 adults + older subjects (73.1% W) (mean age: 56.3 ± 13.8 years; mean BMI: nd)	3 Recall 24 h (every 6 months)	33.1% of TEI SD or SEM: nd
Srour et al., (2020) [87]	France	*n* = 104,707 adults + older subjects (79.2% W) (mean age: 42.7 ± 14.5 years; mean BMI: nd)	3 Recall 24 h via web (every six months; 2 days of the weekend and 1 of the week)	17.3 ± 9.8% of TEI
Beslay et al., (2020) [15]	France	*n* = 110,260 adults + older subjects (78.2% W) (mean age: 43.1 ± 14.6 years; mean BMI: 23.8 ± 4.6 kg/m^2^)	3 Recall 24 h via web (2 week days and 1 day of the weekend; more than 3500 items)	17.1 ± 10.3% of TEI
Vasseur et al., (2020) [88]	France	*n* = 105,832 adults + older subjects (78% W) mean age: 43.3 ± 14.7 years; mean BMI: 23.9 kg/m^2^)	3 Recall 24 h via web (2 week days and 1 day of the weekend; more than 3300 items)	17 ± 9% of TEI SD or SEM: nd
Schnabel et al., (2019) [89]	France	*n* = 44,551 adults + older subjects (73.1% W) (mean age: 56.7 ± 7.5 years; mean BMI: nd)	3 Recall 24 h via web (3000 common foods and drinks)	29.1 ± 10.9% of TEI
Adjibade et al., (2019) [90]	France	*n* = 26,730 adults + older subjects (76.2% W) (mean age 47.2 ± 14.2 years; mean BMI: nd)	3 Recall 24 h (every 6 months; 2 days of the week and 1 day of the weekend)	32 ± 11% of TEI
Schnabel et al., (2018) [91]	France	*n* = 33,343 adults + older subjects (76.4% W) (mean age: 50 ± 14 years; mean BMI: nd)	3 Recall 24 h via web (every 6 months; 3021 common foods and drinks)	33 ± 13.7% of TEI
Rauber et al., (2021a) [36]	UK	*n* = 542 adolescents (% M/W: nd) (mean age: nd; range 11–18 years, mean BMI: nd)	4-day food diary	67.8% of TEI SD or SEM: nd
Rauber et al., (2021b) [47]	UK	*n* = 22,659 adults + older subjects (52.1% W) (mean age: 55.9 ± 7.4 years; mean BMI: 26.7 ± 4.3 kg/m^2^)	4 Recall 24 h via web (200 common foods and drinks)	48.6 ± 17.9% of TEI
Onita et al., (2021) [19]	UK	*n* = 1772 children (51.2% boys) (mean age: nd, range 4–10 years; mean BMI: nd)	4-day food diary	65.4% of TEI SD or SEM: nd
Rauber et al., (2020) [54]	UK	*n* = 6143 adults + older subjects (51.6% W) (mean age: nd, range 19–96 years; mean BMI: nd)	4-day food diary	54.3 ± 0.4% * of TEI
Rauber et al., (2018 and 2019) [46,53]	UK	*n* = 9.374 subjects (% M/W: nd) (mean age: nd, ≥1.5 years; mean BMI: nd)	3–4-day food diary	56.8% of TEI SD or SEM: nd
Adams and White. (2015) [48]	UK	*n* = 2174 adults + older subjects (51.4% W) (mean age: nd, ≥18 years; mean BMI: nd)	4-day food diary	53.1% (95% CI: 52.4–53.7) of TEI
Polsky et al., (2020) [92]	Canada	*n* = 33,924 subjects in 2004 and 20,080 subjects in 2015 (% M/W: nd) (mean age: nd, ≥2 years; mean BMI: nd)	Recall 24 h	2004 years = 47.8% (95% CI: 47.3% to 48.3%) of TEI 2015 years = 45.7% (95% CI: 45.0% to 46.4%) of TEI
Nardocci et al., (2021) [93]	Canada	*n* = 13608 adults + older subjects (% M/W: nd) (mean age: nd, ≥19 years; BMI: nd)	Recall 24 h	47% of TEI SD or SEM: nd
Batal et al., (2018) [94]	Canada	*n* = 3267 adults (62.6% W) (mean age 45.2 ± 14.9 years; mean BMI: nd)	Recall 24 h	54% ±24.6% of TEI
Nardocci et al., (2019) [95]	Canada	*n* = 19,363 adults (50.9% M) (mean age: 45.9 years; mean BMI: 26.9 kg/m^2^)	Recall 24 h	45.1 ± 0.14% * of TEI
Batal et al., (2018) [7]	Canada	*n* = 3700 adults (62.7% W) (mean age: 45.1 years; mean BMI: 30.2 kg/m^2^)	Recall 24 h	53.9% of TEI SD or SEM: nd
Moubarac et al., (2017) [96]	Canada	*n* = 33694 subjects (52.5% F) (mean age: nd, ≥2 years; mean BMI: nd)	2 Recall 24 h	47.7 ± 0.2% * of TEI
Sandoval-Insausti et al., (2020a) [44]	Spain	*n* = 652 older subjects (55.7% M) (mean age: 67.1 ± 5.8 years; mean BMI: nd)	FFQ (860 items)	17.3 ± 10.2% of TEI
Sandoval-Insausti et al., (2020b) [43]	Spain	*n* = 1822 older subjects (51.3% W) (mean age: 68.7 years; mean BMI: nd)	FFQ (860 items)	19.3% of TEI SD or SEM: nd
Da Rocha et al., (2020) [20]	Spain	*n* = 386 children (52% M) (mean age: 5.3 ± 1 years; mean BMI: 15.7 ± 1.6 kg/m^2^)	FFQ (149 items)	32.2 ± 8% of TEI
Blanco-Rojo et al., (2019) [97]	Spain	*n* = 11,898 adults (50.5% W) (mean age: 46.9 ± 0.3 years; mean BMI: nd)	FFQ (880 items)	24.4 ± 0.2% * of TEI
Asma’ et al., (2020) [98]	Malaysia	*n* = 200 adults (75% F) (mean age: 33 years; mean BMI: 25.3 ± 6.8 kg/m^2^)	2 Recall 24 h	24% of TEI SD or SEM: nd
Asma’ et al., (2020) [99]	Malaysia	*n* = 167 adults (74.9% F) (mean age: nd, range 18–49 years; mean BMI = 24.9 ± 5.2 kg/m^2^)	2 Recall 24 h (2 non-consecutive days: 1 of the week and 1 of the weekend)	23% of TEI SD or SEM: nd
Asma’ et al., (2019) [100]	Malaysia	*n* = 200 adults (75% F) (mean age: nd, range 18–59 years; mean BMI: nd)	FFQ (165 items)	40.4% of TEI SD or SEM: nd
Machado et al., (2020a) [101]	Australia	*n* = 7411 adults + older subjects (51.7% M) (mean age: nd, range 20–85 years; mean BMI: 27.4 kg/m^2^)	2 Recall 24 h	38.9% of TEI SD or SEM: nd
Machado et al., (2019 and 2020b) [102,103]	Australia	*n* = 12153 subjects (% M/W: nd) (mean age: nd, ≥2 years; mean BMI: nd)	2 Recall 24 h	42.0% of TEI SD or SEM: nd
Vandevijvere et al., (2020) [104]	Belgium	2004 years, *n* = 3083 subjects (≥15 years; (% M/W: nd) 2014–2015 years, *n* = 3146 subjects (range 3–64 years; 50.8% F)	2 Recall 24 h (adults and teenagers) 2 food diaries for children (3–9 years)	Data from 2004 (survey) = 30.3% (95% CI: 29.3–31.5) of TEI Data from 2014–2015 (survey) = 29.9% (95% CI: 29.0–30.8) of TEI
Khandpur et al., (2020) and Parra et al., (2019) [105,106]	Colombia	*n* = 38,643 adults (51.9% F) (mean age: 26.5 ± 0.2 years; mean BMI: nd)	Recall 24 h	15.9 ± 0.3% * of TEI
Monge et al., (2020) [107]	Mexico	*n* = 64934 adults (100% F) (mean age: 41.7 ± 7.2 years; mean BMI: nd)	FFQ (140 items)	29.8 ± 9.4% of TEI
Marrón-Ponce et al., (2018 and 2019) [108,109]	Mexico	*n* = 10,087 subjects (50.5% W) (mean age: nd, ≥1 years; mean BMI: nd)	Recall 24 h	30.0 ± 4.5% * of TEI
Bonaccio et al., (2021) [55]	Italy	*n* = 24,325 adults + older subjects (% M/W: nd) (mean age: nd, ≥35 years; mean BMI: nd)	FFQ (188 items)	10% of TEI SD or SEM: nd
Dinu et al., (2021) [56]	Italy	*n* = 110 adults (67% F) (mean age: 35.3 ± 9.9 years; mean BMI: 23 ± 3.2 kg/m^2^)	FFQ (94 items)	11 ± 7% of TEI
Shim et al., (2021) [110]	Korea	*n* = 57423 subjects (% M/W: nd) (mean age: nd, ≥1 years; mean BMI: nd)	Recall 24 h	24.9% of TEI SD or SEM: nd Consumption for years: 2010–2012 = 23.1% of TEI 2016–2018 = 26.1% of TEI
Sung et al., (2021) [111]	Korea	*n* = 7364 adults (52.9% M) (mean age: 41.7 ± 0.3 years (M), 42.8 ± 0.3 years (W); mean BMI: nd)	Recall 24 h (every season)	26.8% of TEI SD or SEM: nd
Vedovato et al., (2020) [21]	Portugal	*n* = 1175 children + adolescents (52% M) (mean age: nd, range 4–17 years; mean BMI: nd)	Food diary (1 or 2 week days and 1 day of the weekend)	Data nd (see Table 3)
Costa De Miranda et al., (2021) [16]	Portugal	*n* = 3852 adults + older subjects (% M/W: nd) (mean age: nd, ≥18 years; BMI: nd)	2 Recall 24 h	22.2 ± 0.38% * of TEI
Cediel et al., (2018 and 2020) [112,113]	Chile	*n* = 4920 subjects (60.7% W) (mean age: nd, ≥2 years; mean BMI: nd)	Recall 24 h	28.6 ± 0.5% * of TEI
Fangupo et al., (2021) [22]	New Zealand	*n* = 669 children (% M/W: nd) (mean age: nd, range 12–60 mo.; mean BMI: nd)	FFQ (90 items)	Range: 39.8–54% of TEI
Pinho et al., (2020) [45]	Netherlands	*n* = 8104 older subjects (80.5% W) (mean age 70 ± 10 years; mean BMI 25.8 ± 4.5 kg/m^2^)	FFQ (160 items)	37 ± 11% of TEI
Fliss-Isakov et al., (2020) [114]	Israel	*n* = 652 adults + older subjects (50.8% M) (mean age: 58.5 ± 6.6 years; mean BMI: 28.2 ± 5.4 kg/m^2^)	FFQ (117 items)	38.2 ± 16.2% of TEI
Koiwai et al., (2019) [115]	Japan	*n* = 617 adults (58.5% W) (mean age: 45.6 ± 8.4 years; mean BMI: nd)	Food diary	38.2 ± 0.9% * of TEI
Setyowati et al., (2018) [116]	Indonesia	*n* = 1605 subjects (50.4% W) (mean age: nd, ≥0 years; mean BMI: nd)	Recall 24 h	19.5% of TEI SD or SEM: nd
Nasreddine et al., (2018) [117]	Lebanon	*n* = 302 adults (61.3% W) (mean age: 39.3 ± 13.8 years; mean BMI: nd)	FFQ (80 items)	36.5 ± 16.5% of TEI

Data are reported as mean ± standard deviation (SD) or standard error of the mean (SEM) *; CI, confidence interval; BMI, body mass index; W, women; FFQ, food frequency questionnaire; M, men; mo., months; nd, not determined or reported; UPF, ultra-processed food and drink products; TEI, total energy intake.

Several studies stratified data based on different characteristics, such as age, sex, and BMI. The level of UPF consumption stratified for these parameters is reported in Table 2, Table 3 and Table 4. Slight differences between the sexes were observed in UPF intake, with men having often an overall higher intake compared to women [48,54,87,95,96,111]. However, in most of the studies, the levels of UPF intake appeared comparable [14,30,49,51,81,83,94,112] (Table 2). Regarding age, studies generally reported large variations amongst the age groups. However, UPF intake generally decreased with increasing age, with the highest levels of UPF intake observed in children and adolescents, and the lowest in older subjects [51,54,87,104,108,110,112,118] (Table 3). Only five studies also stratified results based on body mass index (BMI) of the participants, generally finding a slight, but higher, UPF intake in subjects with the highest BMI (e.g., greater than 30 kg/m^2^) [58,87,89,95] (Table 4) [58].

**Table 2:** Level of consumption of ultra-processed foods (UPF) expressed as percent energy provided by UPF intake with respect to total energy intake (TEI) considering sex.

Author (Year)	Sex	UPF Consumption and Statistic
Adams and White (2015) [48]	F = 51.4% M = 48.6%	F = 52.8% (95% CI 51.9–53.7) of TEI M = 53.5% (95% CI 52.3–54.4) of TEI Significantly higher (*p* < 0.05) in men compared to women
Baraldi et al., (2018) [49]	(% M/F: nd)	F = 58.8% (95% CI: 58.1–59.5) of TEI M = 58.3% (95% CI: 57.6–59.0) of TEI No differences between genders
Batal et al., (2018) [94]	F = 62.6% M = 37.4%	F = 54.4 ± 24.4% of TEI M = 53.4± 25.1% of TEI No differences between genders
Bielemann et al., (2015) [58]	F = 48.6% M = 51.4%	F = 51.9% (95% CI: 51.4–52.5) of TEI M = 50.4% (95% CI: 49.9–51.0) of TEI Significantly higher (*p* < 0.001) in women compared to men
Calixto Andrade et al., (2021) [84]	F = 63.3% M = 36.7%	F = 31.4% (95% CI: 30.1–32.7) of TEI M = 30.9% (95% CI: 30.0–31.9) of TEI Statistics: nd
Cediel et al., (2018 and 2020) [112,113]	F = 60.7% M = 39.3%	F = 29.4% (95% CI: 28.1–30.6) of TEI M = 27.8% (95% CI: 26.5–29.2) of TEI No differences between sexes
Da Rocha et al., (2020) [20]	F = 48% M = 52%	F = 32.0% of TEI M = 32.3% of TEI SD or SEM nd Statistics: nd
Gupta et al., (2020) [81]	F = 82.1% M = 17.9%	F = median 59.9% ± 10.8% of TEI M = median 58.4% ± 10.5% of TEI No differences between sexes
Khandpur et al., (2020) [105]	F = 51.9% M = 48.1%	F = 16.2% ± 0.2% * of TEI M = 15.5% ± 0.2% * of TEI Significantly higher (*p* = 0.007) in women compared to men
Machado et al., (2020a) [101]	F = 48.3% M = 51.7%	F = 38.5% of TEI M = 40.7% of TEI SD or SEM nd No differences between sexes
Marrón-Ponce et al., (2018 and 2019) [108,109]	F = 50.5% M = 49.5%	F = 30.1% of TEI M = 29.5% of TEI SD or SEM: nd No differences between sexes
Martínez-Steele et al., (2019) [83]	(% M/F: nd)	F = 55.0 ± 0.5% * of TEI M = 55.9 ± 0.6% * of TEI No differences between sexes
Martinez-Steele et al., (2020) [51]	(% M/F: nd)	F = 58.2 ± 0.5 *% of TEI M = 58.4 ± 0.4 *% of TEI No differences between sexes
Moubarac et al., (2017) [96]	F = 52.5% M = 47.5%	F = 46.5% of TEI M = 48.6% of TEI SD or SEM nd Significantly higher (*p* < 0.001) in men compared to women
Nardocci et al., (2019) [95]	F = 49.1% M = 50.9%	F = 44.2 ± 0.4% * of TEI M = 45.9 ± 0.4% * of TEI Significantly higher (*p* < 0.05) in men compared to women
Rauber et al., (2020) [54]	F = 51.6% M = 48.4%	F = 52.8 ± 0.4% * of TEI M = 55.9 ± 0.6% * of TEI Significantly higher (*p* < 0.05) in men compared to women
Sandoval-Insausti et al., (2020b) [43]	F = 51.3% M = 48.7%	F = 20.7% of TEI M = 17.7% of TEI SD or SEM nd Statistics: nd
Schnabel et al., (2019) [89]	F = 73.1% M = 26.9%	F = 29.4 ± 0.06% * of TEI M = 28.3 ± 0.10% * of TEI Significantly higher (*p* < 0.001) in women compared to men
Shim et al., (2021) [110]	(% M/F: nd)	F = 24.1% (95% CI: 23.8–24.4) of TEI M = 25.8% (95% CI: 25.5–26.1) of TEI Significantly higher (*p* < 0.05) in men compared to women
Simões et al., (2018) [73]	F = 54.2% M = 45.8%	F = 23.0% (IQR: 16.7–29.9) of TEI M = 20.6% (IQR: 14.7–27.5) of TEI Significantly higher (*p* < 0.001) in women compared to men
Smiljanec et al., (2020) [14]	F = 62.5% M = 37.5%	F = 50.8 ± 2.4% of TEI M = 48.8 ± 5.2% of TEI No differences between sexes
Sparrenberger et al., (2015) [30]	F = 50% M = 50%	F = 47.1 ± 1.5% * of TEI M = 49.2 ± 1.6% * of TEI No differences between sexes
Srour et al., (2020) [87]	F = 79.2% M = 20.8%	F = 17.2 ± 9.7% of TEI M = 17.6 ± 9.9% of TEI Significantly higher (*p* < 0.001) in men compared to women
Sung et al., (2021) [111]	F = 47.1% M = 52.9%	F = 25.1 ± 0.38% * of TEI M = 28.4% ± 0.36% * of TEI Significantly higher (*p* < 0.0001) in men compared to women
Vandevijvere et al., (2019) [119]	2004 = (% M/F: nd) 2014–2015 = F = 50.8% M = 49.2%	2004 = F = 28.9% (95% CI: 27.1–30.2) of TEI M = 32.3% (95% CI: 30.9–34.3) of TEI 2014–2015 = F = 29.7% (95% CI: 28.7–31.2) of TEI M = 29.9% (95% CI: 28.6–31.2) of TEI Statistics: nd
Yang et al., (2020) [79]	F = 50.2% M = 49.8%	F = median 54.8% (IQR: 47.8‒61.4) of TEI M = median 55.0% (IQR: 48.4‒61.7) of TEI No differences between sexes

Data are reported as mean ± standard deviation (SD) or standard error of the mean (SEM) *; CI, confidence interval; F, female; M, male; ND, not determined or reported; UPF, ultra-processed food and drink products; TEI, total energy intake.

**Table 3:** Level of consumption of ultra-processed foods (UPF) expressed as percent energy provided by UPF intake with respect to total energy intake (TEI) by considering age.

Author (Year)	Age	UPF Consumption for Age and Statistics
Costa et al., (2021) [60]	*n* = 3128 children (6 years) *n* = 3454 adolescents (11 years)	6 years = 42% (IQR: 34.6–49.8) of TEI 11 years = 32.7% (IQR: 25.1–41.3) of TEI Statistic: nd
Calixto Andrade et al., (2021) [84]	18–39 years = 34.1% 40–59 years = 44.8% >60 years = 21.1%	18–39 years = 39.1% (95% CI: 37.8–40.5) of TEI 40–59 years = 28.1% (95% CI: 27.2–29.0) of TEI >60 years = 21.6% (95% CI: 20.4–22.8) of TEI Statistics: nd
Shim et al., (2021) [110]	nd	1–12 years = 28.9% (95% CI: 28.5–29.4) of TEI 13–19 years = 32.6% (95% CI: 31.9–33.4) of TEI 20–49 years = 27.7% (95% CI: 27.3–28.0) of TEI 50–64 years = 19.6% (95% CI: 19.2–19.9) of TEI >65 years = 15.1% (95% CI: 14.8–15.8) of TEI Significantly higher (*p* < 0.05) in adolescents and lower in subjects older than 65 years
Sung et al., (2021) [111]	*n* = 1114 (19–29 years) *n* = 3301 (30–49 years) *n* = 2949 (50–64 years)	19–29 years = 35.7 ± 0.6% * of TEI 30–49 years = 27.7 ± 0.4% * of TEI 50–64 years = 20.0 ± 0.4% * of TEI Significantly higher (*p* < 0.0001) in younger
Costa De Miranda et al., (2021) [16]	*n* = 3102 (18–65 years) *n* = 750 (>65 years)	18–65 years = 23.8 ± 0.42% * of TEI >65 years = 15.9 ± 0.56% * of TEI Significant differences (*p* = 0.001) between adults and older subjects
Fangupo et al., (2021) [22]	*n* = 501 (12 mo) *n* = 497 (24 mo) *n* = 475 (36 mo)	12 mo = 44.5% (95% CI: 43.0–46.0) of TEI 24 mo = 39.8% (95% CI: 34.6–41.0) of TEI 60 mo = 54% (95% CI: 53.0–54.9) of TEI Intraclass correlation coefficients ranging from 0.23 to 0.36
Leffa et al., (2020) [24]	*n* = 308 children (3 and 6 years)	3 years = 43.4% (IQR 34.3–51.1%) of TEI 6 years = 47.7% (IQR 41.5–53.8%) of TEI Significant differences (*p* < 0.001) between age
Gupta et al., (2020) [81]	*n* = 286 (21–40 years) *n* = 230 (41–50 years) *n* = 239 (≥51 years)	21–40 years = 60.2 ± 11.1% of TEI 41–50 years = 60.6 ± 10.0% of TEI ≥51 years = 58.1 ± 10.9% of TEI Consumption at ≥51 years, but not at 41–50 years, significantly lower (*p* < 0.05) compared to 21–40 years
Martinez Steele et al., (2020) [51]	nd	6–11 years = 68.2 ± 0.5% * of TEI 12–19 years = 66.9 ± 0.7% * of TEI >20 years = 55.9 ± 0.4% * of TEI Significantly lowest (*p* < 0.05) at >20 years
Srour et al., (2020) [87]	*n* = 59,247 (18–44 years) *n* = 28,930 (45–59 years) *n* = 16,530 (>60 years)	18–44 years = 19.4 ± 10.6% of TEI 45–59 years = 14.7 ± 8% of TEI >60 years = 14 ± 7.2% of TEI Significant differences (*p* < 0.001) between groups
Rauber et al., (2020) [54]	19–29 years = 18.7% 30–59 years = 51.0% >60 years = 30.3%	19–29 years = 59.2 ± 1.3% * of TEI 30–59 years = 54 ± 0.4% * of TEI >60 years = 51.8 ± 0.5% * of TEI Significant differences (*p* < 0.05) in the group of subjects aged >60 years
Polsky et al., (2020) [92]	nd	Young children, 2–5 years: 51.0% (95% CI: 49.8 52.3) Children, 6–12 years: 55.8% (95% CI: 55.0–56.6) Adolescent girls, 13–18 years: 57.2% (95% CI: 56.1–58.3) Adolescent boys, 13–18 years: 57.4% (95% CI: 56.2–58.5) Adult girls, 19–54 years: 44.8% (95% CI: 43.8–45.8) Adult men, 19–54 years: 48.2% (95% CI: 47.0–49.4) Older women, >55 years: 41.7% (95% CI: 40.6–42.8) Older men, >55 years: 42.5% (95% CI: 41.5–43.6) 2015 years: Young children, 2–5 years: 48.0% (95% CI: 46.1–49.9) Children 6–12 years: 53.0% (95% CI: 51.9–54.2) Adolescent girls, 13–18 years: 50.4% (95% CI: 48.5–52.4) Adolescent boys, 13–18 years: 53.2% (95% CI: 51.5–54.9) Adult women, 19–54 years: 41.6% (95% CI: 40.2–43.0) Adult men, 19–54 years: 45.4% (95% CI. 43.8–47.0) Older women, >55 years: 45.2% (95% CI: 44.0–46.4) Older men, >55 years: 45.3% (95% CI: 43.9–46.7) Statistic: nd
Machado et al., (2020a) [101]	20–39 years = 38.5% 40–59 years = 36.4% >60 years = 25.1%	20–39 years = 43.4% of TEI 40–59 years = 36.2% of TEI >60 years = 36.2% of TEI SD or SE: nd Statistics: nd
Khandpur et al., (2020) [105]	2–9 years = 18.5% 10–19 years = 23.0% 20–34 years = 26.3% 35–49 years = 20.0% ≥50 years = 12.2%	2–9 years = 19.3 ± 0.3% * of TEI 10–19 years = 19.3 ± 0.3% * of TEI 20–34 years = 15.4 ± 0.3% * of TEI 35–49 years = 12.2 ± 0.3% * of TEI ≥50 years = 11.4 ± 0.4% * of TEI Significant differences (*p* < 0.001) between age groups
Vedovato et al., (2020) [21]	*n* = 1175 children (3 and 7 years)	4 years = 27.3 ± 11.1% of TEI 7 years = 29.3 ± 10.4% of TEI Interclass correlation coefficient = 0.32
Machado et al., (2019 and 2020b) [102,103]	*n* = 822 (2–5 years) *n* = 889 (6–11 years) *n* = 1204 (12–19 years) *n* = 7135 (20–64 years) *n* = 2103 (>65 years)	2–5 years = 47.3% of TEI 6–11 years = 53.1% of TEI 12–19 years = 54.3% of TEI 20–64 years = 39.4% of TEI >65 years = 36.3% of TEI SD or SE: nd Statistic: nd
Vandevijvere et al., (2019) and (2020) [104,119]	*n* = 992 (3–9 years) *n* = 928 (10–17 years) *n* = 1226 (18–64 years)	3–9 years = 33.3% (95% CI: 32.1–35.0) of TEI 10–17 years = 29.2% (95% CI: 27.7–30.3) of TEI 18–64 years = 29.6% (28.5–30.7) of TEI Significantly higher (*p* < 0.05) in children compared to adolescents and adults
Neri et al., (2019) [18]	*n* = 2411 (2–5 years) *n* = 3335 (6–11 years) *n* = 3726 (12–19 years)	2–5 years = 58.2% of TEI 6–11 years = 66.2% of TEI 12–19 years = 66.4% of TEI SD or SE: nd Statistics: nd
Martínez-Steele et al., (2019) [83]	*n* = 2126 (20–39 years) *n* = 2239 (40–59 years) *n* = 2020 (>60 years)	20–39 years = 58.9 ± 0.6% * of TEI 40–59 years = 54.6 ± 0.8% * of TEI >60 years = 52.2 ± 0.6% * of TEI Significantly lower (*p* < 0.001) at >60 years compared to 40–59 and 20–39 years
Schnabel et al., (2019) [89]	nd	45–64 years = 29.6 ± 0.06% * of TEI ≥65 years = 26.3 ± 0.13% * of TEI Significant differences (*p* < 0.001) between groups
Nardocci et al., (2019) [95]	nd	18–34 years = 50.2 ± 0.55% * of TEI 35–44 years = 20.6 ± 0.77% * of TEI 45–64 years = 34.9% ± 0.46% * of TEI >65 years = 41.9 ± 0.42% * of TEI Significantly lower (*p* < 0.05) at 35–55 years, 45–64 years, and >65 years compared to 18–34 years
Cediel et al., (2018 and 2020) [112,113]	*n* = 1374 (2–19 years) *n* = 1668 (20–49 years) *n* = 948 (50–64 years) *n* = 930 (>65 years)	2–19 years = 38.6% (95% CI: 35.7–39.4) of TEI 20–49 years = 26.7% (95% CI: 26.2–29.1) of TEI 50–64 years = 21.8% (95% CI: 19.5–23.6) of TEI >65 years = 18.3% (95% CI: 15.9–18.9) of TEI Significantly lower (*p* < 0.001) at >65 years
Marrón-Ponce et al., (2018 and 2019) [108,109]	1–4 years = 7.6% 5–11 years = 16.1% 12–19 years = 14.5% >20 years = 61.8%	1–4 years = 38.6% of TEI 5–11 years = 34.3% of TEI 12–19 years = 35.5% of TEI ≥20 years = 26.2% of TEI SD or SEM: nd Significant differences (*p* < 0.05) between age groups
Rauber et al., (2018 and 2019) [46,53]	nd	1.5–10 years = 63.5 ± 0.34% * of TEI 11–18 years = 68.0 ± 0.40% * of TEI 19–64 years = 54.9 ± 0.35% * of TEI >65 years = 53.0 ± 0.52% * of TEI Significant differences (*p* < 0.001) in children and adolescents
Setyowati et al., (2018) [116]	0–4 years = 6.5% 5–12 years = 14.1% 13–18 years = 11.5% 19–55 years = 55% >55 years = 12.9%	0–4 years = 41.4% of TEI 5–12 years = 29% of TEI 13–18 years = 27.8% of TEI 19–55 years = 16.1% of TEI >55 years = 9% of TEI SD or SE: nd Statistic: nd
Simões et al., (2018) [73]	35–44 years = 22.3% 45–54 years = 39.4% 55–64 years = 27.9% 65–74 years = 10.4%	35–44 years = 24.8% (IQR: 18.6–31.5) of TEI 45–54 years = 22.2% (IQR 16.3–29.0) of TEI 55–64 years = 20% (IQR 14.1–26.7) of TEI 65–74 years = 19.5% (IQR 13.5–26.4) of TEI Significantly higher (*p* < 0.001) in the group aged 35–44 years and then decreasing with age
Baraldi et al., (2018) [49]	nd	2–9 years = 65.9% (95% CI: 65.0–66.8) of TEI 10–19 years = 66.8% (95% CI: 65.9–67.7) of TEI 20–39 years = 59.5 % (95% CI: 58.7–60.3) of TEI 40–59 years = 55.2% (95% CI: 54.1–56.4) of TEI >60 years = 52.8 % (95% CI: 51.9 -53.7) of TEI Significantly lowest (*p* < 0.05) at >60 years
Moubarac et al., (2017) [96]	*n* = 13,779 (2–18 years) *n* = 3812 (19–30 years) *n* = 5601 (31–50 years) *n* = 4611 (51–64 years) *n* = 5891 (>65 years)	2–18 years = 55.1% of TEI 19–30 years = 51% of TEI 31–50 years = 44.9% of TEI 51–64 years = 42.4% of TEI >65 years = 42.6% of TEI SD or SE: nd Significantly higher (*p* < 0.001) at >65 years
Karnopp et al., (2017) [29]	<24 mo = 72.5% >24 mo = 27.5%	<24 mo = 19.7 ± 1.3% * of TEI >24 mo = 36 ± 0.8% * of TEI Statistics: nd
Sparrenberger et al., (2015) [30]	*n* = 66 (55% F) (2–6 years) *n* = 36 (43.4% F) (7–10 years)	2–6 years = 43.7 ± 1.4% * of TEI 7–10 years = 54.7 ± 1.7% * of TEI Significant differences (*p* < 0.001) between groups
Adams and White (2015) [48]	18–29 years = 19.3% 30–39 years = 17.0% 40–49 years = 19.0% 50–59 years = 15.7% 60–69 years = 13.8% ≥70 years = 15.2%	18–29 years = 58.2% (95% CI: 56.3–60.2) of TEI 30–39 years = 55.9 % (95% CI: 54.5–57.3) of TEI 40–49 years = 52.5% (95% CI: 50.7–53.6) of TEI 50–59 years = 49.7% (95% CI: 48.1–51.3) of TEI 60–69 years = 49% (95% CI: 47.5–50.5) of TEI ≥70 years = 50.6% (95% CI: 49.0–52.2) of TEI Significant negative association between age and percentage of TEI from UPF

Data are reported as mean ± standard deviation (SD) or standard error of the mean (SEM) *; CI, confidence interval; mo, months; ND, not determined or reported; UPF, ultra-processed food and drink products; TEI, total energy intake.

**Table 4:** Level of consumption of ultra-processed foods (UPF) expressed as % energy provided by UPF intake with respect to total energy intake (TEI) by considering the BMI factor.

Author (Year)	Body Mass Index (BMI)	UPF Consumption for BMI and Statistics
Bielemann et al., (2015) [58]	BMI <24.9 kg/m^2^ = 70.8% BMI 25–29.9 kg/m^2^ = 20.8% BMI ≥30 kg/m^2^ = 8.4%	BMI 25–29.9 kg/m^2^ = 50.5% of TEI BMI <24.9 kg/m^2^ = 51.6% of TEI SD or SEM nd Significant differences (*p* = 0.003) between groups
Nardocci et al., (2019) [95]	BMI 18.5–24.9 kg/m^2^ = 40.2% BMI 25–29.9 kg/m^2^ = 37.6% BMI ≥30 kg/m^2^ = 22.2%	BMI 18.5–24.9 kg/m^2^ = 44.3 ± 0.4% * of TEI BMI 25–29.9 kg/m^2^ = 44.8 ± 0.45% * of TEI BMI ≥30 kg/m^2^ = 46.8 ± 0.6% * of TEI Significantly higher (*p* < 0.05) in obese subjects
Schnabel et al., (2019) [89]	nd	BMI <18.5 kg/m^2^ = 28.3 ± 0.30% * of TEI BMI 18.5–24.9 kg/m^2^ = 28.6 ± 0.07% * of TEI BMI 25–29.9 kg/m^2^ = 29.3 ± 0.10% * of TEI BMI ≥30 kg/m^2^ = 31.3 ± 0.16% * of TEI Significant differences (*p* < 0.001) between groups
Srour et al., (2020) [87]	BMI <25 kg/m^2^ = 69.1% BMI 25–29.9 kg/m^2^ = 20.2% BMI ≥30 kg/m^2^ = 7.8%	BMI <25 kg/m^2^ = 17.1 ± 9.7 % of TEI BMI 25–29.9 kg/m^2^ = 17 ± 9.6% of TEI BMI ≥30 kg/m^2^ = 18.8 ± 11.1% of TEI Significant differences (*p* < 0.001) between groups
Vandevijvere et al., (2019) [119]	BMI 18.5–24.9 kg/m^2^ = 40.2% BMI 25–29.9 kg/m^2^ = 37.6% BMI ≥30 kg/m^2^ = 22.2%	BMI 18.5–24.9 kg/m^2^ = 30.7% (95% CI: 29.1–31.9) of TEI BMI 25–29.9 kg/m^2^ = 28.5% (95% CI: 27.5–31.1) of TEI BMI ≥30 kg/m^2^ = 29.3% (95% CI: 26.6–31.1%) of TEI No significant differences between groups

Data are reported as mean ± standard deviation (SD) or standard error of the mean (SEM) *; CI, confidence interval; BMI, body mass index; ND, not determined or reported; UPF, ultra-processed food and drink products; TEI, total energy intake.

Two studies evaluated the intake of UPF during pregnancy, reporting conflicting results. Silva et al. [37] reported no difference between second and third trimester. Conversely, Gomes et al. [34] reported a significant difference in the second and third trimesters between the intervention and control group. This latter consumed more UPF compared to the intervention group that received training for the application of healthy food practices during prenatal care appointments (see Supplementary Table S1). Finally, Gehring and colleagues [85,86] compared UPF intake in subjects adhering to different dietary patterns, observing a higher UPF intake in vegans and vegetarians than pesco-vegetarians and meat eaters (Supplementary Table S1).

## 4. Discussion

Since Monteiro et al. proposed the NOVA classification to categorize foods based on the degree of processing [4], several studies have been conducted to estimate the level of consumption of UPF and its association with several health markers [24,35,52,67,120] as well as with disease risk and mortality [55,59,87,121,122], adjusting the models for energy intake and other potential confounding factors. It was hypothesized that a high level of UPF consumption may represent a health issue, being associated with weight gain and worsening of cardiovascular risk factors such as high waist circumference and low HDL cholesterol [11]. 

In the present study, we collected 100 unique studies published in 106 manuscripts estimating the UPF levels in different populations from 21 countries around the world. Overall, we found a large variability in the percent of TEI obtained from UPF in the different countries, with the United States and United Kingdom being the countries with the highest percent of TEI from UPF, and Mediterranean countries such as Italy showing the lowest level (~10% of TEI). These results are in line with previous evidence suggesting that adherence to the Mediterranean diet is inversely associated with UPF consumption [20]. This is further confirmed by findings showing that the highest tertiles or quartiles of UPF intake are associated with the lowest adherence to the Mediterranean diet [44,55]. The low levels of UPF consumption registered in Italy and other Mediterranean countries are those associated with the lowest risks for non-communicable diseases. For instance, da Silva et al. recently observed an association between UPF consumption and the increased presence of high waist circumference, overweight, and peripheral arterial disease when comparing the third and first tertiles of the UPF contribution to energy intake in a Brazilian cohort [123]. Intriguingly, the first tertile corresponded to <10.6% of TEI from UPF, which is similar to the levels registered in the study conducted in the Italian population. These levels are far lower than those detected in the first quartiles in the study by Rauber et al., who observed that participants in the highest quartile (>70.3% and >71.7% of TEI from UPF in women and men, respectively) had a significantly higher risk of developing obesity, and of experiencing a ≥5% increase in BMI waist circumference than those belonging to the lowest quartile (<24.1% and <26.3% of TEI from UPF in women and men, respectively) [47]. The type of foods contributing to UPF intake largely varied among countries, but, in accordance with previous findings [124], the most consumed UPF included: baked goods, dairy products, processed fruit and vegetables, and, among drinks, carbonated drinks. 

In addition to country, the level of UPF intake was found to be inversely associated with the increase in age. In this regard, children generally showed the highest intake of UPF, which led the European Childhood Obesity Group to “a call to action” aimed to inform people about the potential harmful effects of UPF [118]. For example, it was found that in the United Kingdom, 65% of calories eaten by primary and secondary school children derived from white bread, biscuits, carbonated drinks, crisps, and chips. These findings are in line with the observations reported by others [125,126]. Similarly, the pediatric populations of the United States and Canada reported an intake of UPF above 55% by including breads, cookies, savory snacks, reconstituted meat products, milk-based drinks, breakfast cereals, juices and sodas, and frozen and ready-to-eat meals in the diet [18,96]. Among U.S. school-aged children and adolescents, UPF provided 66.2% and 66.4% of TEI, respectively, with pizzas, sodas, and juices being the most-consumed products [118]. In this scenario, it was proposed that the levels of UPF intake in the young may reflect, at least in part, their lifestyle. In this regard, a recent study documented that subjects consume more UPF when dining out than when eating at home [127]. Other reasons are related to socio-economic inequalities, including lower education status of the mother or unemployed parents, which may lead to a preference for cheaper and less nutritious foods [105]. A different trend was observed for older subjects who showed a lower intake of UPF compared to younger subjects; the main UPF products included cookies and pastries, but also processed breads, breakfast cereals, and yogurts [16,128]. Compared to age, a minor variability was found for sex and/or BMI, which might differ for the net amount of UPF consumption but not for the percent of TEI from UPF. Intriguingly, the adherence to specific dietary patterns represented an additional determinant of the levels of UPF consumption. In this context, based on the NOVA classification, vegans and vegetarians reported higher UPF consumption compared to pesco-vegetarians and meat eaters, mainly driven by a higher consumption of plant-based meat and dairy substitutes. These results highlight the high variability in the characteristics of these types of diet, which may differ widely for the consumption of several food groups [129]. However, these results were found only in two different publications belonging to the same cross-sectional trial performed in the NutriNet-Santé cohort [85,86]; thus, a confirmation of such an analysis deserves further investigation, also to comprehend better if all vegetarian diets have the same health benefits regardless of the levels of UPF consumed. 

A thorough comparison of the findings from the different studies considered was challenging due to the differences in food classification and to the disparate definitions that were proposed. Descriptions of UPF within the NOVA system vary with distinguishing features including single vs. 2–3 vs. ≥5 more ingredients, or natural/fresh vs. imitation or industrial, and whole foods vs. fractioned substances [13]. This means that different studies may have classified the same food as UPF or not based on the distinguishing feature used for classifying foods.

Notably, one of the main sources of variation among study protocols is the tools used for estimating UPF intake. Overall, from the present review, it is difficult to provide conclusive findings about the influence of the tools used to determine UPF intake since its estimation was typically performed using a single method. In this regard, most of the studies (*n* = 49) used the 24 h recall. To reflect the typical diet, this tool needs to be administered several times; however, in some studies, data were derived from a single 24 h recall, which could have affected the significance of the findings. However, this tool has the strength of being able to assess the consumption of all food items since subjects can include/report specific information (e.g., brand), which may help with identifying the NOVA group. However, a lower number of studies assessed UPF intake using FFQs, which are not always specifically created and validated to estimate the consumption of products undergoing different food processing, and the food list cannot cover all the food items consumed, thus leading to underreporting [130]. Among studies using FFQs, some of them used the questionnaire developed within the European Prospective Investigation into Cancer and Nutrition (EPIC) [131], which is not able to distinguish among products belonging to different NOVA groups. This is, for instance, the case for artisanal or industrial breads or cakes that belong to two different NOVA groups (Groups 3 and 4, respectively). Therefore, the use of tools not specifically validated for estimating UPF consumption may potentially lead to the misclassification of foods in the UPF categories, which, in turn, may lead to the misinterpretation of the associations found with markers of health. Only two studies [17,51] used a questionnaire specifically validated for estimating the levels of UPF consumption in children and adults. The approach to validating and using ad hoc developed FFQs, when possible, created for specific populations to consider the different dietary habits, should be recommended for more accurately estimating the consumption of UPF and the actual impact on health-related outcomes.

This study has some strengths worth highlighting, the first of which is the rigorous search and selection strategy that identified available studies examining the energy intake from UPF. However, we cannot exclude that the use of further databases may have allowed the identification of additional studies. Secondly, food processing level was always determined according to the criteria of NOVA classification, to facilitate the comparison amongst findings, although it was reported that some NOVA definitions are open to researchers’ interpretation [132], leading to a not-always-uniform categorization [133]. Finally, we included only studies reporting results as the percent of TEI from UPF and not as grams per day. In our opinion, the ratio of energy intake from UPF compared to total energy intake is more useful for reflecting the impact of these products on the whole diet. However, this method may not be useful for detecting the consumption of energy-free products (such as energy-free drinks with artificial sweeteners); thus, this choice can also be considered as a limitation. Notably, the NOVA system used has been largely criticized for different reasons, mostly because this system focuses on the role of food processing, regardless of the nutritional characteristics of foods, on human health [132]. Thus, further efforts should be directed toward elucidating the impact of the different foods belonging to the same NOVA category but with different nutrient profiles on human health. To conclude, it seems worthwhile to implement a critical and constructive discussion able to clarify the potential and applicability of this type of approach.

## 5. Conclusions

In conclusion, this review showed high levels of UPF consumption, especially in some countries and in specific target groups (i.e., children and adolescents). However, most of the data on UPF consumption have been derived from FFQs and 24 h dietary recall, which are not specifically validated for estimating UPF; thus, such data should be considered with caution. In this scenario, tools specifically validated to estimate the levels of UPF consumption can be useful to avoid misinterpretation of the findings, especially when used to investigate the association with health status. Despite several studies reporting a positive association between UPF and obesity and cardiometabolic health, on the whole, the evidence is not yet totally convincing. In addition, whether this association is dependent on the nutritional characteristics of UPF and/or related to the applied processing is unclear. In this context, despite the NOVA system classifying foods based on the food processing technology without providing any information about the nutritional content of the food, the UPF group has been suggested to be an indicator of poor food quality due to the generally high amounts of free or added sugars, fats, low levels of fiber, and high energy density. For instance, it was recently observed that UPF consumption is associated with a deterioration in diet quality, with UPF intake being negatively correlated with fiber and protein and positively correlated with sugar, fat, and saturated fat intake [115]. This highlights that the concept of UPF may be somehow misleading, with the effect on human health mediated more by the nutritional quality of products rather than the processing. The association between UPF and nutritional adequacy is not surprising, since, for instance, the presence of added sugar or fat is a major element in defining UPF. Finally, the classification systems based on processing are not often aligned with dietary guidelines (e.g., some products considered UPF are recommended within a balanced diet). Thus, confirmation of the results already obtained should be accompanied by an evaluation of the association between UPF consumption and health status, estimating the contribution within different dietary patterns.

## Data Availability

The data presented in this study are available on request from the corresponding author.

## Supplementary Materials

The following are available online at https://www.mdpi.com/article/10.3390/nu13082778/s1, Supplementary Table S1. Level of consumption of ultra-processed foods (UPF) expressed as percent energy provided by UPF intake in the total energy intake (TEI) in pregnant women and following different dietary patterns.
