# Supplementary material for: A Systematic Review of Worldwide Consumption of Ultra-Processed Foods: Findings and Criticisms
Source: Nutrients. 2021 Aug 13;13(8):2778. doi: 10.3390/nu13082778 (PMC8398521; doi:10.3390/nu13082778)
Supplement: Supplementary file 1 [file nutrients-13-02778-s001.zip › nutrients-1301497-supplementary.pdf]

Supplementary Table 1 –Level of consumption of ultra-processed foods (UPF) expressed as % energy provided by UPF intake with respect to total energy intake (TEI) in pregnant women and following different dietary patterns

Author (year)	Country	Study population	UPF consumption and statistics
**PREGNANTS**			
Silva et al., (2021) [37]	Brazil	n = 42 pregnant women with pregestational diabetes mellitus (100% F)<br><br>(mean age 31.5 ± 5.8 y; mean BMI: nd)	Second trimester = 16.9 ± 7.7% of TEI<br>Third trimester = 15.2 ± 10% of TEI<br>No difference between trimesters
Gomes et al. (2019) [34]	Brazil	n = 353 pregnant women (100% F)<br><br>(mean age: nd; mean BMI: nd)	First trimester = 23.9% vs. 26% of TEI (intervention group vs control group, respectively)<br>No difference between group<br>Second trimester = 20.6% vs 27.3% of TEI (intervention group vs control group, respectively)<br>Significantly different (p<0.001) between groups<br>Third trimester = 22.8% vs. 26.7% of TEI (intervention group vs control group, respectively)<br>Significantly different (p=0.022) between groups<br><br>SD or SEM nor reported
**DIETARY PATTERNS**			
Gehring et al., (2020 and 2021) [64,65]	France	n = 21212 subjects (73.1% F)<br>(mean age 56.3 ± 13.8 y; mean BMI: nd)<br>19812 meat eaters<br>646 pesco-vegetarians<br>500 vegetarians<br>254 vegans	Meat eaters = 33% of TEI<br>Pesco-vegetarians = 32.5% of TEI<br>Vegetarians =37% of TEI<br>Vegans = 39.5% of TEI<br>SD or SEM not reported.<br>Significantly (p<0.001) higher in vegetarian and vegans compared to the other dietary patterns

*Data are reported as mean ± standard deviation (SD) or standard error of the mean (SEM)**

CI, confidence interval; BMI: body mass index; ND, not determined; UPF, ultra-processed food and drink products; y: year; TEI, total energy intake
